# Down-Regulation of Gli Transcription Factor Leads to the Inhibition of Migration and Invasion of Ovarian Cancer Cells via Integrin β4-Mediated FAK Signaling

**DOI:** 10.1371/journal.pone.0088386

**Published:** 2014-02-12

**Authors:** Qi Chen, Rong Xu, Chunyan Zeng, Quqin Lu, Dengliang Huang, Chao Shi, Weilong Zhang, Libin Deng, Runwei Yan, Hai Rao, Guolan Gao, Shiwen Luo

**Affiliations:** 1 Department of Obstetrics and Gynecology, The Second Affiliated Hospital of Nanchang University, Nanchang, Jiangxi, China; 2 Department of Biostatistics & Epidemiology, School of Public Health, Nanchang University, Nanchang, Jiangxi, China; 3 Center for Experimental Medicine, The First Affiliated Hospital of Nanchang University, Nanchang, Jiangxi, China; 4 Institute of Translational Medicine, Nanchang University, Nanchang, Jiangxi, China; 5 Department of Molecular Medicine, University of Texas Health Science Center, San Antonio, Texas, United States of America; 6 Department of Obstetrics and Gynecology, General Hospital of Beijing Aeronautics, Beijing, China; Aix-Marseille University, France

## Abstract

**Background:**

Recent evidence suggests that aberrant activation of Hedgehog (Hh) signaling by Gli transcription factors is characteristic of a variety of aggressive human carcinomas including ovarian cancer. Therefore, chemotherapeutic agents that inhibit activation of Gli transcription factors have emerged as promising novel therapeutic drugs for ovarian cancer.

**Results:**

In this study, we show that activation of Hh signaling promoted cellular migration and invasion, whereas blockade of Hh signaling with GANT61 suppressed cellular migration and invasion in ovarian cancer cells. After treatment with GANT61, cDNA microarray analyses revealed changes in many genes such as Integrin β4 subunit (ITGB4), focal adhesion kinase (FAK), etc. Furthermore, ITGB4 expression was up-regulated by Sonic Hedgehog (Shh) ligand and down-regulated by Hh signaling inhibitor. The Shh-mediated ovarian cell migration and invasion was blocked by neutralizing antibodies to ITGB4. In addition, phosphorylations of FAK were increased by Shh and decreased by Hh signaling inhibitor. Inhibition of Gli1 expression using siRNA mimicked the effects of GANT61 treatment, supporting the specificity of GANT61. Further investigations showed that activation of FAK was required for Shh-mediated cell migration and invasion. Finally, we found that down-regulation of Gli reduced the expression of ITGB4 and the phosphorylated FAK, resulting in the inhibition of tumor growth *in vivo*.

**Conclusions:**

The Hh signaling pathway induces cell migration and invasion through ITGB4-mediated activation of FAK in ovarian cancer. Our findings suggest that the diminishment of crosstalk between phosphorylated FAK and ITGB4 due to the down-regulation of Gli family transcription factors might play a pivotal role for inhibiting ovarian cancer progression.

## Introduction

Ovarian cancer is one of the most lethal gynecologic malignancies worldwide. Due to the non-specific symptoms, most of ovarian cancer cases present with advanced stage disease and are associated with a high mortality rate [1]. In advanced ovarian cancer, tumor cells are highly invasive and subsequent cancer metastases leads to death [2]. Deregulated cell migration and invasion contribute to the attainment of the metastatic ability of the tumor cells and the molecular mechanism that regulates metastasis remains largely unknown. Therefore, it is crucial to identify molecular mediators that confer metastatic potential to ovarian cancer cells and eventually to use those molecules as biomarkers for predicting risk of ovarian cancer progression [3].

Hedgehog (Hh) signaling is initiated by the binding of ligand namely Sonic Hedgehog (Shh), Indian Hedgehog (Ihh) or Desert Hedgehog (Dhh) to its receptor, Patched (Ptch) which thereby, diminishes the inhibitory effects of Ptch on Smoothened (Smo) [4]. Smo is then localized into the primary cilium, an organelle playing a critical role in Hh signaling [5] as evidence from Smo mediated activation of an intracellular cascade in cilium that results in nuclear translocation of Gli2, a Gli family transcription factor [6]. In turn, translocated Gli2 induces the transcription of Hh target genes such as Gli1, a crucial component of Hh signaling [6], [7]. The ability of these Gli transcription factors to turn on genes in the nucleus can indeed promote cellular proliferation, cellular survival, stemness, and cell fate determination in a variety of organs [8], [9]. More importantly, Hh-driven cancers arise from a variety of mutations that affect different components, including the key transcriptional effectors Gli proteins [10]–[12]. Constitutively, Hh-Gli signaling is hyperactive in basal cell carcinomas, medulloblastomas and cancers of esophagus, due to mutation in Patched or Smoothened [13]. Also, the presence of deregulated Hh-Gli signaling axis was observed in melanomas and prostate carcinomas [14], [15]. Furthermore, several reports indicate that Hh signaling is involved in the metastases of ovarian [16], prostatic [17], gastric [18], esophageal [19] and pancreatic [20], [21] carcinomas. Together, these observations imply that disregulated Hh signaling correlates with the severity of the associated tumor and contributes to maintaining metastatic behavior. Nevertheless, the role of Hh signaling in invasion and metastasis of ovarian cancer remains ill-defined. In addition, the mechanisms by which Hh signaling promotes tumor invasion and metastasis need to be further elucidated.

There are many indications that the Hh signaling pathway is essential for the growth of cancer cells. For this reason, the inhibition of Hh signaling by new therapeutic agents has been attempted in multiple human cancers [22]–[24]. Blockade of Hh signaling by GDC-0449 [24], [25], cyclopamine [24], IPI-926 [23], or Smo shRNA [26] inhibited cell proliferation and survival, and suppressed tumor formation. However, a number of tumors have been shown to be refractory to the direct effects of pharmacologic Hh inhibition with Smo antagonists due to natural or acquired mutations in Smo [27], [28] or amplification of downstream effector Gli2 [29]. These observations highlight the need for identifying better therapeutic targets that will effectively block the Hh signaling pathway. GANT61 (Gli-ANTagonist 61) was identified from a cell-based screen for inhibitors of Gli-mediated transcription [29]. Recently, GANT61 has been identified as a potent Hh signaling pathway inhibitor for treating various cancers [22], [24].

In this study, we employed GANT61, a small molecule inhibitor of both Gli1 and Gli2 [29], to identify unique downstream targets of the *GLI* genes that function specifically in cellular migration and invasion in ovarian cancer. Our results obtained from human ovarian cancer cell lines SKOV3 cells, which exhibits high invasive behavior [30], support the Hh signaling promotes cancer cell invasion through integrin β4 (ITGB4)-mediated activation of focal adhesion kinase (FAK) in ovarian cancer. In fact growing evidence suggests that ITGB4 plays a pivotal role in functions associated with carcinoma progression [31]–[33]. Interestingly, FAK has been linked to integrin-signaling pathways via interactions with integrin-associated proteins such as paxillin and talin [34]–[37] with resultant effects on cell migration [37], [38]. Moreover, in mouse xenograft models of human ovarian cancer, inhibition of the Hh signaling pathway can promote extensive cell death and reduce tumor growth *in vivo*. Our findings suggest that the diminishment of crosstalk between phosphorylated FAK and ITGB4 due to the down-regulation of Gli family transcription factors might play a pivotal role for the inhibition of ovarian cancer progression.

## Results

### Down-regulation of Gli expression impairs ovarian cancer cell migration and invasion

**Figure 1:**
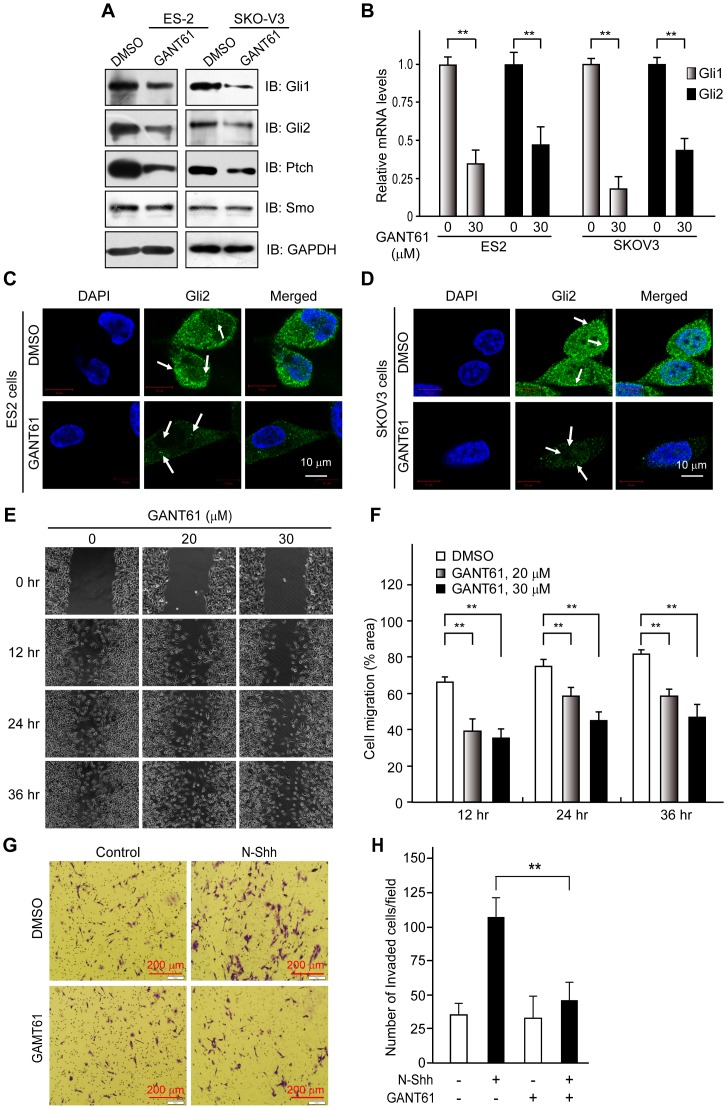
Down-regulation of Gli decreased ovarian cancer cell migration and invasion. (**A**) Exposure to GANT61 (30 µM; 48 hr) reduces expression of both Gli1 and Gli2 protein, determined by Western blot. GAPDH was used as the loading control. (**B**) Expression of both Gli1 and Gli2 mRNA is decreased following treatment with GANT61 (30 µM; 48 hr), determined by real-time PCR. (**C, D**) GANT61 inhibits expression of Gli2 in ovarian cancer cells. ES2 and SKOV3 cells were treated with control vehicle (DMSO) or GANT61 (20 µM) for 48 hr. Subsequently, cells were stained with Gli2 and visualized under a confocal microscope. Scale bar, 10 µm. (**E, F**) Blockade of Hh signaling inhibits ovarian cancer cell migration. Confluent ES2 cell monolayers were wounded with a pipette tip and then treated with N-Shh conditional medium plus control vehicle (0.2% DMSO) or GANT61. Cell migration to the wound area was monitored by microscopy. The percentage of total area covered by cells was then assessed using the NIH Image program. (**G, H**) Blockade of Hh signaling inhibits ovarian cancer cell invasion. SKOV3 cells (1×10^5^ cells/well) were seeded in the upper chamber. Growth medium containing N-Shh (0.5 µg/ml) alone or together with GANT61 (20 µM), control vehicle (0.2% DMSO) were added to the lower chamber. After 24 hr of incubation, cells that invaded the lower surface of the insert were stained with crystal violet and counted by microscopy. The representative images from three independent experiments are shown. Scale bar, 200 µm. The data are expressed as mean ± SD for experiments performed three times. **, *P<*0.01, compared with control groups.

Previous studies have suggested that aberrant activation of Hh signaling is implicated in a variety of aggressive human carcinomas [10], [12], [16]. In this study, we first examined the effects of GANT61, a specific inhibitor of Gli1 and Gli2 [29], [39], on the Hh signaling pathway by measuring the expression of Hh receptors (Ptch and Smo) and effectors (Gli1 and Gli2). GANT61 inhibited the expression of transcription factor Gli1 and Gli2 (**Figures 1A and B**). Similarly, GANT61 inhibited the expression of Ptch, a downstream target of Gli [40]. Also, we employed immunofluorescence technique to examine the effects of GANT61 on the expression of Gli. Immunofluorescence image showed GANT61 inhibited the expression of Gli2 (**Figures 1C and D**). These data suggest that GANT61 can inhibit the Hh signaling pathway.

Next, we tested the effects of Hh signaling on the motility of human ovarian cancer cells using an *in vitro* wound-healing assay. Two human ovarian cancer cell lines ES2 and SKOV3 were treated with the conditional medium containing N-Shh (0.5 µg/ml) and the control medium. We found that N-Shh significantly enhanced ES2 and SKOV3 cell migration (data not shown). To confirm the contribution of Hh signaling to the motility of ovarian cancer cells, the cells were treated with an inhibitor of the Hh signaling pathway. The additional incubation of N-Shh-treated cells with increasing concentrations of GANT61 reversed the stimulatory effect of N-Shh on cell migration in ES2 cells, versus cells treated with N-Shh plus control vehicle (**Figures 1E and F**), suggesting that GANT61 inhibited ES2 cell migration. Furthermore, the effect of Hh signaling on the invasive ability of ovarian cancer cells was measured using a Matrigel invasion assay. The ability of ovarian cancer cells to invade Matrigel was markedly enhanced by treatment with Shh (**Figures 1G and H**). Conversely, the Shh-induced invasiveness of SKOV3 cells was reduced by nearly 64% in cells that were also treated with GANT61 (**Figures 1G and H**), suggesting that Hh signaling has an essential role in the motility of ovarian cancer cells.

### Inhibition of Hh signaling alters gene expression profiles of ovarian cancer cells

**Figure 2:**
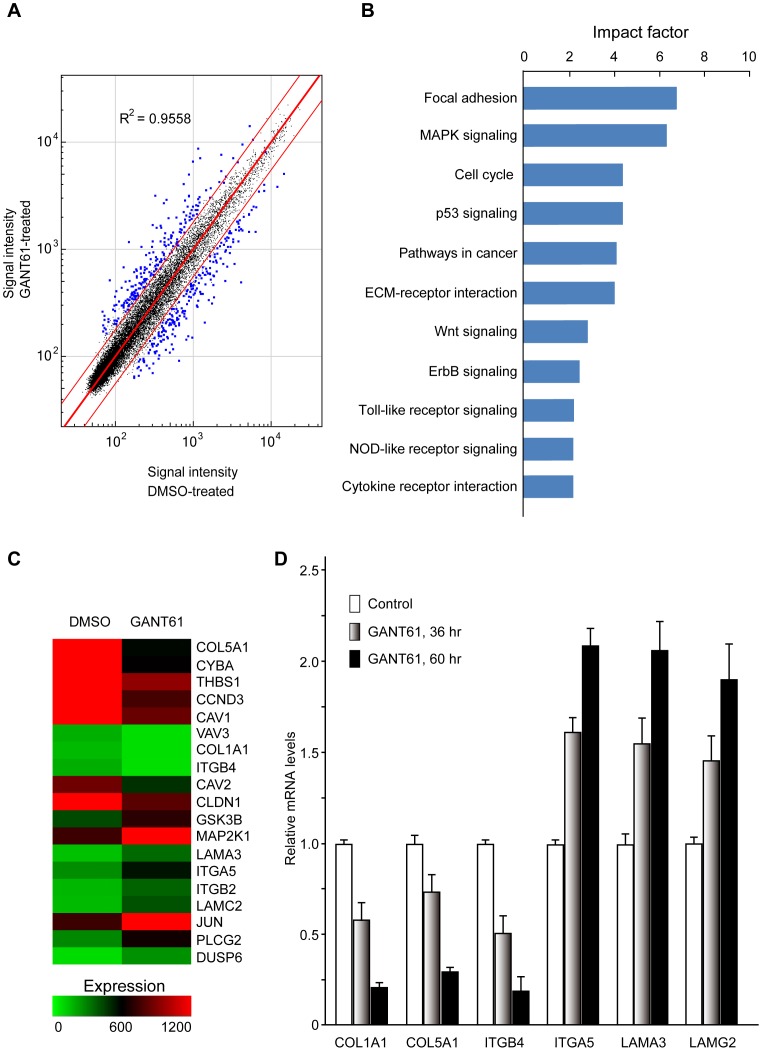
Gene expression profiles in GANT61-treated SKOV3 cells. (**A**) Identification of differentially expressed genes (DEGs) in GANT61-treated SKOV3 cells. Cells were treated with GANT61 (20 µM) or DMSO for 60 hr, and total RNA was extracted as described in Methods. Changes in gene expression were determined by cDNA microarray gene profiling using the Illumina Human HT expression BeadChip V4 (Illumina Inc., San Diego, CA). Differential expressions for the DEGs are shown. (**B**) The top 11 canonical signaling pathways influenced by inhibition of Gli1/Gli2 function in SKOV3 cells. The top 11 canonical signaling pathways, determined by IPA, that were significantly up-regulated or down-regulated by GANT61 treatment in SKOV3 cells, are shown. The 412 DEGs were mapped to the IPA-defined network. The significant *P*-values that determine the probability that the association between the genes in the dataset and the canonical pathway is by chance alone were calculated by Fisher's exact test, and are expressed as −log (*P*-value). (**C**) Heat map of DEGs in the focal adhesion signal pathway in GANT61-treated SKOV3 cells. The heat map shows that 17 genes were significantly differentially expressed, including seven up-regulated genes and 12 down-regulated genes, in the GANT61-treated compared to control SKOV3 cells. (**D**) Selected DEGs from cDNA array gene expression profiling analyzed by real-time PCR. SKOV3 cells were treated with DMSO or GANT61 for the indicated times, total RNA was extracted for real-time PCR as described in Methods using the primer sets in **Table 1**. The data represent the mean ± SD of three determinations, and GAPDH was used to normalize the relative mRNA levels.

To investigate the role of the Hh signaling pathway in the initiation and progression of ovarian cancer, we measured gene expression levels in response to inhibition of Hh signaling in ovarian cancer cells using a cDNA microarray technique. SKOV3 cells were treated with either 20 µM GANT61 or DMSO as vehicle control for 60 hr. Then, we compared the gene expression profiles of GANT61-treated SKOV3 cells and DMSO-treated cells with Illumina® Sentrix® BeadChip arrays. The expression of 18,401 human genes was profiled in control cells treated with vehicle and in cells treated with GANT61. Genes with a *DiffScore* less than −20 or more than 20 (i.e. *P*-value<0.01) were considered Differentially Expressed Genes (DEGs) induced by GANT61 relative to the vehicle control. Of the 412 DEGs unique to SKOV3 (20 µM GANT61 for 60 hr), 208 genes (50.5%) were up-regulated and 204 genes (49.5%) were down-regulated (**Figure 2A**). And, almost all of *DEGs* (392/412) showed a considerable expression change after GANT61-treatment (fold change >2.0). Genes with significant changes in expression following GANT61 treatment were classified into different categories based on well-documented and established biological or pathological function (**Figure 2B**). These DEGs in response to treatment with GANT61 mainly belong to the following categories: focal adhesion, MAPK signaling, cell cycle, p53 signaling, extracellular matrix (ECM)-receptor interaction, Wnt signaling, ErbB signaling, Toll-like receptor signaling, NOD-like receptor signaling and cytokine receptor interaction. DEGs operating in the focal adhesion in GANT61-treated cells are presented in a heat map (**Figure 2C**). Through this map, we found that 19 genes were significantly differentially expressed, including seven up-regulated genes and 12 down-regulated genes, compared to control SKOV3 cells. Interestingly, some DEGs observed in the focal adhesion such as LAMC2, ITGA5, LAMA3, ITGB4, COL1A1, THBS1 and COL5A1 were also found among the DEGs in the ECM-receptor interaction. These findings suggest that the focal adhesion and ECM-receptor interaction cross-talk in SKOV3 cells after treatment with GANT61, and the expression change of “focal adhesion” -related genes plays an important role in response to GANT61-treatment.

To determine the robustness of cDNA microarray gene expression profiling following treatment of SKOV3 cells with GANT61, real-time PCR was employed to determine changes in expression of the selected group of six DEGs identified from the cDNA microarrays. The involved genes and synthesized primers are shown in **Table 1**. Real-time PCR was performed on cDNA that was generated by using total RNA independently isolated from GANT61-treated SKOV3 cells for 0 hr, 36 hr, and 60 hr. GAPDH was used to normalize all real-time PCR data (**Figure 2D**). The changes determined by real-time PCR were found to be consistent with the microarray analysis, thus validating its significance. Overall, these data indicate that the Hh signaling pathway may play a pivotal role in the motility and invasiveness of ovarian cancer cells.

**Table 1:** Primers used for real-time PCR amplification.

Genes	Forward primer (5′ to 3′)	Reverse primer (5′ to 3′)
Gli1	5′-TCCTACCAGAGTCCCAAGTT-3′	5′-CCCTATGTGAAGCCCTATTT-3′
Gli2	5′-CCTGGCATGACTACCACTATGAG-3′	5′-GGCTTGGCTGGCATGTTG-3′
LAMA3	5′-CTGTCACTCGGCGGTATT-3′	5′-TGTGGTGCTGGCATTCA-3′
COL1A1	5′-GTGCGATGACGTGATCTGTGA-3′	5′-TGGTCGGTGGGTGACTCTG-3′
COL5A1	5′-GGCTGGGAAGGAAGAGGAC-3′	5′-CATACGGTGAGGGCGTGTAG-3′
ITGA5	5′-CTCAACAACTCGCAAAGCG-3′	5′-TGGGAATAGCACTGCCTCA-3′
ITGB4	5′-GCGACTACACTATTGGATTTGGC-3′	5′-TGTCAGGCTGATGACGTTCTTG-3′
LAMC2	5′-GAGTGAGAACCACCAACCG-3′	5′-GGCAGGAGGAGCGAGAA-3′
FAK	5′-CATCCCTAACCATTGCG-3′	5′-GCCCGTTCACCTTCTTT-3′
GAPDH	5′-CAGGGCTGCTTTTAACTCTGGT-3′	5′-GATTTTGGAGGGATCTCGCT-3′

### ITGB4 mediates Shh-induced migration and invasion of ovarian cancer cells

**Figure 3:**
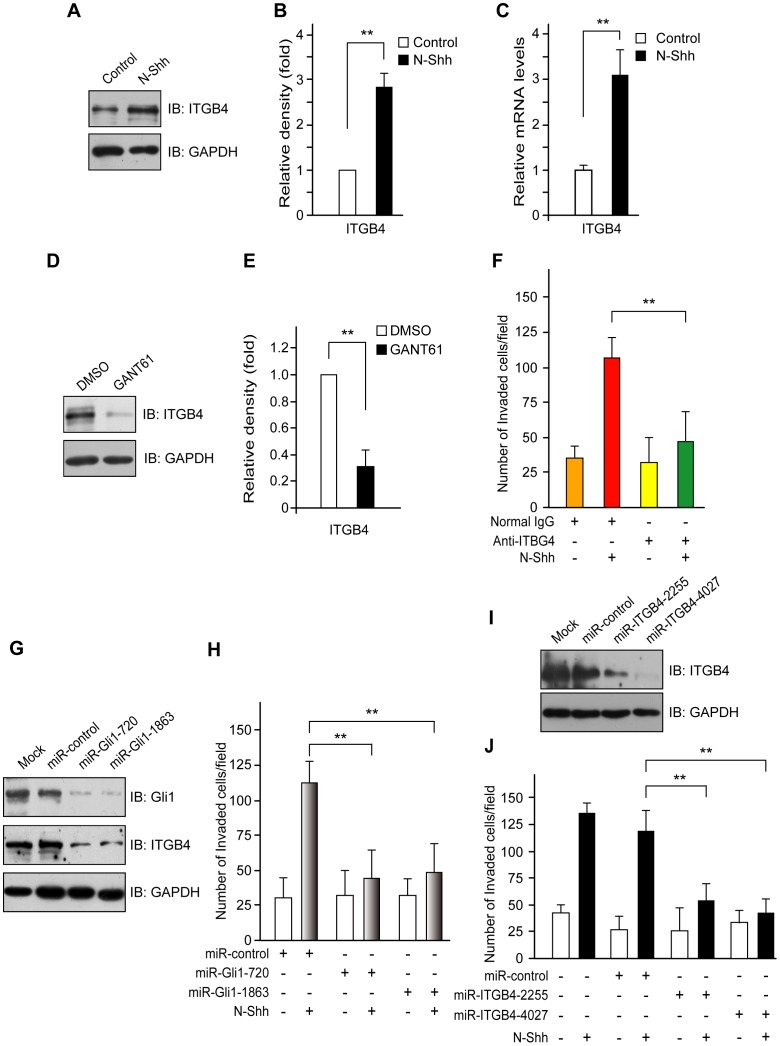
ITGB4 mediates Shh-induced ovarian cancer cell invasion. (**A, B**) Stimulation of N-Shh up-regulates ITGB4 expression. SKOV3 cells were incubated in the presence or absence of N-Shh (0.5 µg/ml) for 24 hr. Western blots were then performed for the indicated antibodies. Protein expression was quantified by Image J and normalized to GAPDH. (**C, D**) Inhibition of Gli1 down-regulates ITGB4 expression. SKOV3 cells were treated with GANT61 or DMSO for 48 hr. Western blot was then performed with the indicated antibodies. Protein expression was quantified by Image J and normalized to GAPDH. (**E, F**) Blockade of ITGB4 inhibits cell invasion in SKOV3 cells. SKOV3 cells grown in low serum (2% FBS) growth medium were seeded into the upper chambers. The low serum growth medium containing N-Shh (0.5 µg/ml) together with anti-ITGB4 antibody (0.2 µg/ml) or control IgG was added to the lower chamber. After 24 hr of incubation, cells that invaded the lower surface of the insert were stained with crystal violet and counted by microscopy. (**G**) Gli1 miRNAi inhibits the expression of ITGB4 in SKOV3 cells. SKOV3 cells were transiently transfected with vehicle (lipofectamine alone), control miRNAi and Gli1 miRNAis (miR-Gli1-720 and miR-Gli1-1863), respectively. Western blot showed that Gli1 miRNAi inhibited the expression of ITGB4 genes. (**H**) Silencing Gli1 expression impairs Shh-induced ovarian cancer cell invasion. SKOV3 cells were transfected with Gli1 miRNAi or control miRNAi for 24 hr followed by stimulation with N-Shh (0.5 µg/ml) or control medium for another 24 hr. Cell invasion was measured as described in Methods. (**I, J**) Silencing ITGB4 expression impairs Shh-induced ovarian cancer cell invasion. SKOV3 cells were transfected with ITGB4 miRNAi constructs (miR-ITGB4-2255 and miR-ITGB4-4027) or control miRNAi for 24 hr followed by stimulation with N-Shh (0.5 µg/ml) or control medium for another 24 hr. Western blot showed that ITGB4 miRNAis inhibited the expression of ITGB4 gene (**I**). Cell invasion was measured as described in Methods (**J**). The data are expressed as mean ± SD for experiments performed three times. **, *P<*0.01, compared with control groups.

Numerous studies have shown that ITGB4-FAK signaling might modulate the metastatic potential of ovarian cancer cells by breaking down basement membrane barriers and promoting cell motility [41]–[43]. To determine if Shh-stimulated cell migration and invasion resulted from elevated levels of ITGB4 protein, we examined the expression level of ITGB4 using Western blot analysis and real-time PCR. The ITGB4 expression was increased after N-Shh stimulation, with levels 3-fold greater than untreated cells being observed after 24 hr (**Figures 3A, B and C**). In contrast, ITGB4 expression was markedly reduced in the presence of GANT61 (**Figures 3D and E**). To determine if abrogation of ITGB4 signaling could block the Shh-stimulated cellular motility and invasion, we blocked ITGB4 signaling by using an anti-ITGB4 blocking antibody. As shown in **Figure 3F**, the stimulatory effects of N-Shh on cell migration and invasion were completely abolished by anti-ITGB4 blocking antibody, but not by normal IgG.

To confirm the role of transcription factor Gli in mediating ITGB4 expression, we transfected SKOV3 cells with miRNAi constructs targeting the Gli1. Immunoblot analysis in **Figure 3G** showed miR-Gli1-720 and miR-Gli1-1863, which reduced the expression of Gli1 by 5- and 4-fold, respectively, significantly decreased ITGB4 subunit expression. Next, we evaluated the effects of direct silencing of Gli1 expression on cell behavior. As shown in **Figure 3H**, a significant decrease in cell invasion was observed in Gli1 miRNAi-treated cells as assessed by the cell invasion assay. Finally, we sought to determine whether ITGB4 contributes to the invasive phenotype mediated by the Hh signaling. For these experiments, we utilized highly invasive ovarian cancer cells SKOV3 [30]. ITGB4 expression was suppressed using specific miRNAi constructs, and the Shh-stimulated cellular invasion of treated cells to non-targeting (miR-control) or untreated cells was compared. The loss of ITGB4 expression (**Figure 3I**) reduced the Shh-stimulated cellular invasion of SKOV3 cells by ∼60% compared with untreated or non-targeting (miR-control) miRNAi–transfected cells (**Figure 3J**), suggesting that ITGB4 is important for tumor cell invasion.

### Shh promotes motility and invasiveness of ovarian cancer cells through ITGB4-mediated activation of FAK signaling

**Figure 4:**
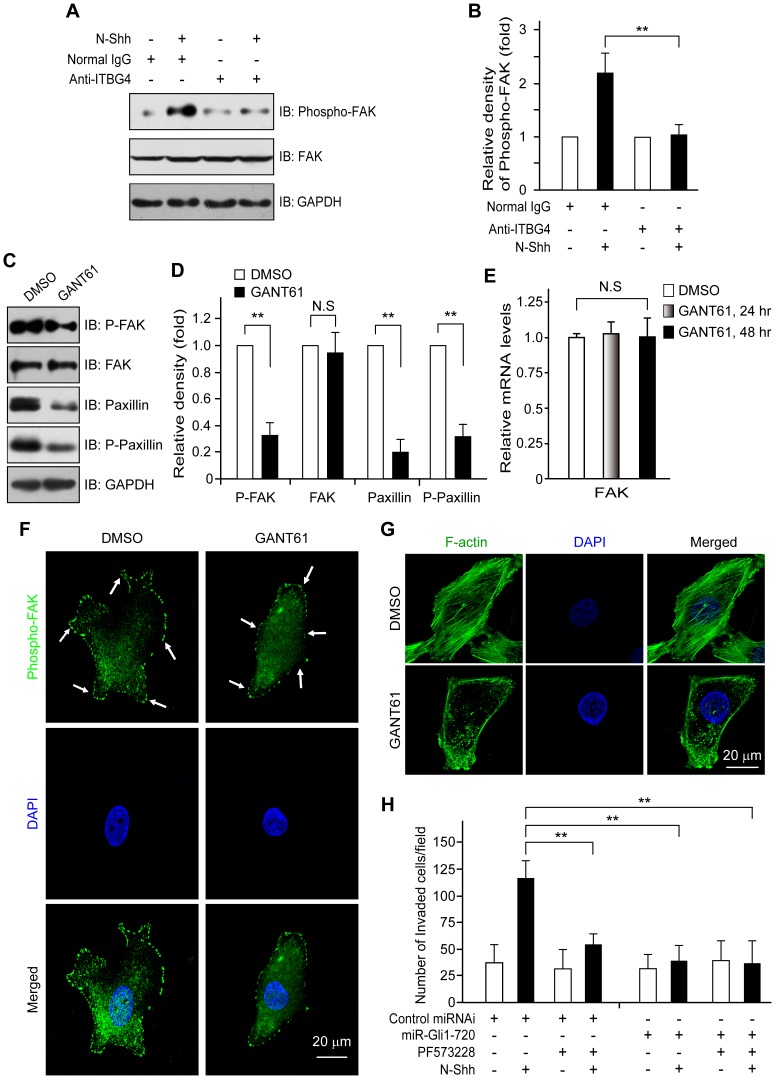
ITGB4-FAK signaling mediates Shh-induced cellular migration and invasion. (**A, B**) Hh signaling activates FAK through ITGB4. SKOV3 cells were treated with ITGB4 antibody or control IgG in the presence or absence of N-Shh (0.5 µg/ml) for 24 hr. Expression and phosphorylation of FAK (Tyr397) were verified by Western blot. (**C–E**) Blockade of Gli down-regulates phosphorylation levels of FAK (Tyr397) but not FAK protein expression. SKOV3 cells were treated with GANT61 or DMSO for 24 hr. Then Western blot was performed using the indicated antibodies. N.S, no significance. (**F, G**) Repression Gli expression decreases the phosphorylation of FAK (Tyr397) and cytoskeletal organization. SKOV3 cells were treated with GANT61 for 48 hr. Subsequently, cells were stained with antibody against phosphorylation of FAK (Tyr397) and visualized using confocal microscope (**F**). For F-Actin staining, cells were incubated with Alexa Fluor 488 phalloidin (Invitrogen, A12379) for 20 min and visualized using confocal microscope (**G**). (**H**) Loss of Gli-FAK signaling impairs Shh-induced ovarian cancer cell invasion. SKOV3 cells were transfected with control miRNAi or Gli1 miRNAi (miR-Gli1-720) for 24 hr followed by stimulation with N-Shh (0.5 µg/ml) together with PF573228 (5 µM), a specific inhibitor of FAK, or control vehicle (DMSO) for another 24 hr. Cell invasion was measured by invasion assay as described in Methods. The data are expressed as mean ± SD for experiments performed three times. **, *P<*0.01, compared with control groups. Scale bar, 20 µm.

To elucidate the molecular mechanisms underlying the Shh-stimulated ITGB4 response in cellular motility and invasion of ovarian cancer cells, we evaluated FAK protein expression and activation by ITGB4 to determine if they were affected by Hh signaling. As shown in **Figure 4A**, phosphorylation of FAK (Tyr397) was found to be significantly enhanced as a result of N-Shh stimulation. However, in spite of the presence of N-Shh, treating the SKOV3 cells with anti-ITGB4 blocking antibody significantly decreased phosphorylation of FAK (Tyr397) (**Figures 4A and B**). In addition, stimulating SKOV3 cells with increasing concentrations of N-Shh revealed that the expression of ITGB4 and phosphorylation of FAK (Tyr397) increased in a dose-dependent manner (data not shown). Furthermore, the phosphorylation of FAK (Tyr397) was reduced in cells that were cotreated with GANT61 when compared with cells treated with N-Shh plus control vehicle (**Figures 4C, D and F**). Reprobing with an antibody directed against total FAK did not reveal any significant change in protein and mRNA levels (**Figures 4C, D and E**), suggesting that the observed increases in expression did not occur as a result of an increase in the amount of total FAK protein, which is consistent with the results that the blockade of Hh signaling reduced the expression of ITGB4 (**Figures 3D and G**). Interestingly, when the Hh signaling pathway was blocked using GANT61, a reduced expression of paxillin, which is downstream of FAK [44], was observed (**Figure 4C and D**). Similarly, inhibition of Hh signaling resulted in the disruption of cytoskeletal organization in ovarian cancer cells (**Figure 4G**). These findings indicate that Hh signaling plays a critical role in the induction of FAK phosphorylation and the cytoskeletal organization.

To further evaluate the role of ITGB4-FAK signaling in Shh-stimulated cellular migration and invasion, we also inhibited and knocked down FAK in SKOV3 cells and analyzed for cellular motility and invasion. Importantly, additional treatment with PF573228, a specific inhibitor of FAK [45], completely reversed the motile and invasive effects of Hh signaling on SKOV3 cells (**Figure 4H**). Furthermore, transient expression of FAK miRNAi reduced the ability of N-Shh to stimulate migration and invasion of those cells compared with the cells treated with control miRNAi (data not shown). Of note, the reduction of FAK expression with a specific miRNAi construct used here was shown previously [46]. Taken together, these findings demonstrate that Hh signaling modulates the migration and invasion of ovarian cancer cells through ITGB4-mediated activation of FAK signaling.

### Blockade of Hh signaling inhibits ovarian cancer growth *in vivo*

**Figure 5:**
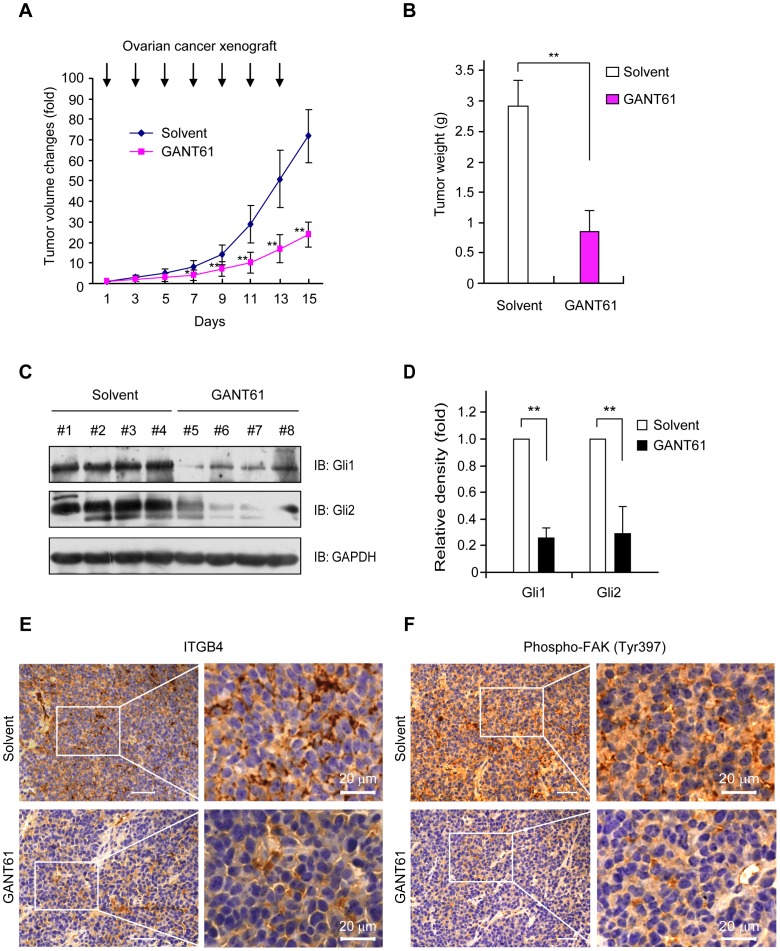
Effect of Gli blockade on growth of human ovarian cancer cells *in vivo*. SKOV3 cells (2×10^7^) were implanted s.c. into the flank of BALB/c nu/nu mice. Afterwards the mice were received either GANT61 (25 mg/kg s.c., thrice per week) or solvent (corn oil: ethanol, 4∶1). Treatment was initiated in all groups (n = 8 each group) when mean tumor volume had reached 100 mm^3^. (**A**) Treatment with GANT61 led to a significant growth inhibition of xenografted human ovarian cancer tumors. n = 8; **, *P*<0.01. (**B**) The experiment was terminated on day 15, and tumors were excised. Final tumor weights in the GANT61 treatment group (n = 8) were significantly lower compared with excised tumors of the solvent control group (n = 8). **, *P*<0.01. Columns, mean; bars, SD. (**C, D**) Western blot analyses for Gli1 and Gli2 in tumor tissues. Treatment with GANT61 diminished expression of Gli1 and Gli2. (**E, F**) Immunohistochemical image of tumor tissue section. Treatment with GANT61 diminished expression of ITGB4 (**E**). A substantial decrease in phosphorylation of FAK (Tyr397) was also effectively achieved by GANT61 treatment (**F**). Scale bar, 20 µm.

To estimate the potential growth inhibitory effects of GANT61 *in vivo*, we subsequently implanted SKOV3 cells s.c. into mice. Treatment with GANT61 (25 mg/kg, thrice per week) significantly reduced the growth of tumors compared with the solvent control (**Figure 5A**). This was also reflected by final tumor weights on day 15, which were significantly lower in the GANT61 treated group (**Figure 5B**). We also investigated whether Gli could be down-regulated by this treatment. Western blot analyses of tumor tissues revealed that treatment with GANT61 indeed down-regulated Gli1 and Gli2 in tissues (**Figure 5C and D**). In addition, the intensity of both ITGB4 and phosphorylation of FAK (Tyr397) was significantly reduced in tumor sections of the GANT61 group compared to the solvent control group (**Figure 5E and F**). This result suggests that down-regulation of Gli reduces the expression of ITGB4 and the phosphorylated FAK, resulting in the inhibition of tumor growth *in vivo*.

## Discussion

Although the critical role of Hh signaling in tumorigenesis has been highlighted [47]–[50], the molecular mechanisms by which the Hh signaling pathway mediates metastasis is not well understood. Here, we report that the Hh signaling pathway promotes ovarian cancer cell migration and invasion *in vitro* and *in vivo*. Moreover, we found that the activation of the Hh signaling pathway induced ITGB4 expression and activation of FAK. Finally, we showed that disruption of ITGB4-FAK signaling resulted in suppression of Hh-mediated cell migration and invasion. Based on these findings, we suggest that the Hh signaling pathway induces metastatic functions of ovarian cancer through ITGB4-mediated activation of FAK.

Previous studies have suggested that Hh signaling plays a role in the control of the motility of multiple cell types [51]–[53]. Disruption of Hh signaling by cyclopamine has been shown to inhibit EMT and reduce metastasis in pancreatic cancer cells [20]. Gli overexpression induced lymphatic metastasis, whereas the inhibition of Hh signaling reduced cell growth and motility [19]. In this study, we showed that the migration and invasion of ovarian cancer cells were induced by N-Shh stimulation and blocked by abrogating Hh signaling with GANT61 (**Figure 1**). Interestingly, we also found that activation of the Hh signaling pathway in ovarian cancer cells induced ITGB4 gene transcription (**Figure 3**). Furthermore, blockade of ITGB4 signaling by an ITGB4-blocking antibody renders ovarian cancer cells unresponsive to Shh-stimulated migration and invasion, suggesting that the ITGB4 modulates Hh signaling in ovarian cancer migration and invasion (**Figure 3**). Combined, these data indicate that abnormal activation of this signaling may contribute to metastasis in a variety of cancers, including ovarian cancer, and that inhibition of the Hh signaling pathway may be useful for preventing metastasis. It is interesting to note that enhancement of ITGB4 gene expression was induced by N-Shh stimulation (**Figure 3**). Therefore, Hh signaling needs to be further evaluated to determine if it affects the activity of the ITGB4 promoter through a physical interaction between the Gli protein and a specific region of the ITGB4 promoter.

Accumulating evidence indicates that integrins can alter cellular behavior through the recruitment and activation of signaling proteins such as non-receptor tyrosine kinases, including FAK and c-Src that form a dual kinase complex [54]. The FAK-Src complex binds to and can phosphorylate various adaptor proteins such as p130C and paxillin [55]. Activation of FAK by both integrin and growth factors plays a critical role in a variety of biological processes, including cell survival, proliferation, attachment, migration and invasion [56]–[58]. Overexpression of FAK protein has been reported in metastatic human colorectal, breast, prostate, and ovarian cancer cells [59]–[62]. In addition, elevated expression levels of both FAK and phosphorylated FAK are correlated with malignant hepatoma invasion and metastasis [63]–[65]. However, most of these findings were obtained with normal fibroblasts, and thus it is unclear whether Integrin/FAK functions in a similar manner in human tumor cells, especially ovarian carcinoma. To better understand the mechanism by which ITGB4 involved Shh-stimulated migration and invasion, we evaluated these effects to determine if expression and activation of FAK were mediated through ITGB4. We found that Shh stimulation of ovarian cancer cells induced phosphorylation of FAK (Tyr397), but not expression of FAK, whereas treatment with GANT61 or an ITGB4-blocking antibody resulted in a significant decrease in phosphorylation of FAK (Tyr397) (**Figure 4**). Furthermore, additional treatment with PF573228, a specific inhibitor of FAK [45], or knock down of FAK using FAK miRNAi constructs [46], completely reversed the motile and invasive effects of Hh signaling. Taken together, these results strongly suggest that the motile and invasive activities of Hh signaling on ovarian cancer cells can be attributed to ITGB4-mediated activation of FAK. However, the precise mechanism by which the Hh signaling pathway controls ITGB4–FAK function to regulate Shh-mediated metastatic potential needs to be further investigated.

Consistent with previous reports that FAK is activated by ITGB4 and mediates ITGB4-dependent cell motility [66], our data also suggest that ITGB4 is an essential mediator of FAK activation and cancer cell motility. The novel aspect of our study is the finding that Hh signaling through ITGB4 activates FAK. Indeed, recent reports suggests that FAK is a key downstream effector of ITGB4 [31], [32], [67], [68] and is involved in ITGB4-dependent anchorage- independent growth [33], [34]. Although the exact mechanism by which FAK mediates ITGB4-dependent signaling remains to be determined, our study suggests that FAK mediates ITGB4-dependent cell motility in SKOV3 cells. As shown by our study (**Figure 4**), FAK is likely to play a major role in regulating ITGB4-dependent cell motility, but other Src family kinase isoform(s) could be involved as well. The identity of the Src kinase isoform(s) that mediates ITGB4 signaling is currently under investigation using siRNA-dependent selective knockdown.

**Figure 6:**
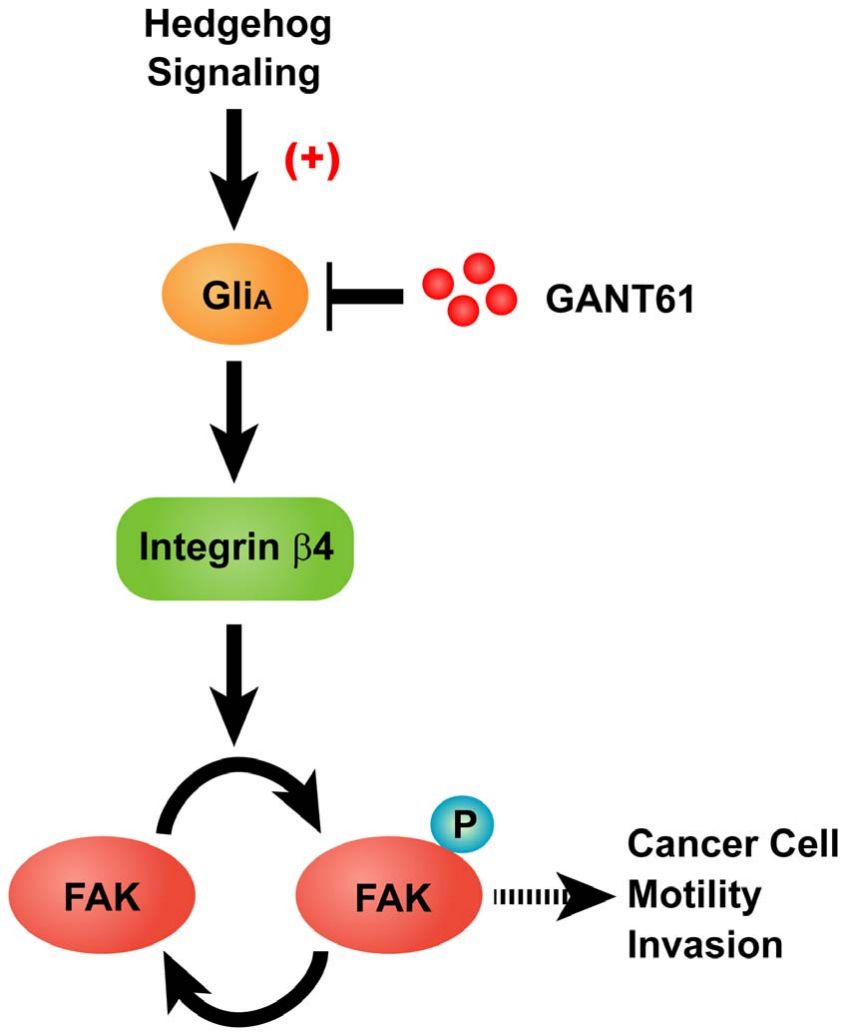
Hh signaling regulates ovarian cancer cell migration and invasion *via* integrin β4-mediated FAK signaling. Proposed role of Hh signaling activation involving ITGB4/FAK dependent signaling pathway.

As previously reported, the level of FAK activation correlates with metastatic potential [60]–[63]. However, the mechanism that regulates activation of FAK in aggressive cancer cells has not been elucidated. These studies, taken together with our current data, strongly suggest that increased activation of FAK by ITGB4 could be associated with the development of metastasis. Future studies will involve experimental as well as spontaneous metastasis assays to determine the contribution of FAK activation to ITGB4-dependent carcinoma progression. We will also examine whether the level of ITGB4 *in vivo* correlates with activation of FAK in tumors by performing tissue microarray. Based on previous studies and our data, we speculate that aberrant activation of Hh signaling pathway triggers the cell migration/invasion in ovarian cancer cells *via* ITGB4-mediated FAK signaling (**Figure 6**). However, this molecular model may be more complexed with the participation of additional signaling proteins, such as Src, PI3K/AKT, and Wnt.

In summary, our findings emphasize the potential role of ITGB4–FAK signaling in Shh-stimulated cellular migration and invasion. In addition, we presented experimental evidence that strongly supports the role of Gli down-regulation as anti-tumor in ovarian cancer. These observations reveal a novel molecular mechanism responsible for the Hh signaling-mediated ovarian cancer cell migration and invasion and a potentially valid therapeutic target for the treatment of ovarian cancer.

## Methods

### Reagents and antibodies

GANT61 (G9048), protease inhibitor cocktail and Lubrol-PX were purchased from Sigma-Aldrich (St. Louis, MO). PF573228 (sc-204179) was purchased from Santa Cruz (Dallas, TX). The primary antibodies were purchased from Cell Signaling (Gli1, 2648S; Phospho-Paxillin (Tyr118), 2541); Abcam (Smo, ab38686; Ptch, ab55629; Gli2, ab26056; ITGB4, ab77801); BD (FAK(Tyr397), 611722); Millipore (Paxillin, 05-417; GAPDH, mAb374); Santa Cruz (FAK, sc-558; normal IgG, sc-2025). Enhanced chemiluminescence (ECL) western blot detection reagents were from Thermo Fisher Scientific Inc. (Rockford, IL). BLOCK-iT Pol II miR RNAi Expression Vector Kit (K4936-00), Lipofectamine 2000 (11668-019), Alexa Fluor 488 donkey anti-mouse IgG (A21202) and Alexa Fluor 488 phalloidin (A12379) were purchased from Invitrogen (Cambridge, MA). Other chemicals used were of analytical grade purchased from Sigma-Aldrich (St. Louis, MO).

### Cell culture

Ovarian carcinoma cell lines namely ES2 and SKOV3 were purchased from the American Type Culture Collection (ATCC, Manassas, VA). Cells were cultured in RPMI-1640 medium supplemented with 10% FBS, penicillin (100 U/ml), and streptomycin (100 µg/ml). Transient transfection of cells was performed with lipofectamine 2000 (Invitrogen, 11668-019) according to the instructions of the manufacturer (Invitrogen, Carlsbad, CA). In all experiments, the medium was replaced daily.

### Expression plasmids and N-Shh conditional medium

Gli1- and ITGB4-miRNAi expression vectors were generated using the BLOCK-iT™ Pol II miR RNAi Expression Vector Kit (K4936-00, Invitrogen, Carlsbad, CA) [46], [69]. The oligonucleotide sequences for miRNAi constructs were as follows: for miR-Gli1-720 (targeting nucleotides 720 to 740 of Gli1; GenBank accession no. NM_005269), 5′-TTC ATA CAC AGA TTC AGG CTC-3′; for miR-Gli1-1863 (targeting nucleotides 1863 to 1883 of Gli1), 5′-AAG ACC ACC TAT CCG ATC CAG-3′; for miR-ITGB4-2255 (targeting nucleotides 2255 to 2275 of ITGB4; GenBank accession no. NM_000213.3), 5′-CTT AAA GCC CAC CAT GTG ACC-3′; and for miR-ITGB4-4027 (targeting nucleotides 4027 to 4047 of ITGB4), 5′-TAC ATA AGG AAG CTG TCG TAG-3′. The numbers in the miRNAi constructs indicate the start of targeted nucleotide sequences of the gene. A secretary expression plasmid encoding Flag-tagged Shh-N-terminal peptide was constructed by inserting the amino-terminal signaling domain of a human Shh (aa 26-184) cDNA (GenBank accession no. NM_000193.2) [70] into a pFlag-CMV1 vector (Sigma-Aldrich, E7273). For N-Shh conditional medium (N-Shh), HEK293T cells were transfected with10 µg of the Flag-N-Shh plasmid. Twelve hours after transfection, the media were replaced with DMEM medium supplemented with 5% FBS. The cells were incubated for an additional 24 hr. After that, the media were harvested and stored at 4°C until used [71].

### Immunofluorescence and immunohistochemistry

Cells were fixed in 4% paraformaldehyde and incubated at 4°C overnight with primary antibodies in PBS containing 0.5% goat serum and 0.1% Triton X-100. After washing three times with PBS, samples were incubated at room temperature for 1 hr with Alexa Fluor 488 donkey anti-mouse IgG. Isotype-specific negative controls were included with each staining. For F-Actin staining, cells were incubated with Alexa Fluor 488 phalloidin (Invitrogen, A12379) in PBS containing 1% BSA for 20 min at room temperature. Representative photographs were taken by a Zeiss 700 confocal microscope. The immunohistochemical staining was performed as described previously [72]. Images were captured with a FSX100 microscope (Olympus).

### Western blot analysis

Following the designed program, cells were harvested after treatment with GANT61 or DMSO and subjected to Western blot analysis as described previously [46], [69]. Briefly, cells were solubilized in the extraction buffer containing 0.5% Lubrol-PX, 50 mM KCl, 2 mM CaCl_2_, 20% glycerol, 50 mM Tris-HCl, and inhibitors of proteases and phosphatases, pH 7.4. Cell lysates were cleaned by centrifugation at 12,000 rpm for 15 min and assayed for protein concentration by using a standard BCA protein assay kit (23225, Thermo). Lysates were subjected to SDS-PAGE and subsequent immunoblotting with antibodies. Quantification of immunoblots was done by scanning films containing nonsaturated signals with Epson V700 scanner. All experiments were repeated at least three times with consistent results.

### Cell migration and invasion assays

Cell migration was measured using a scratch assay [73]. ES2 cells were plated onto 6-well plates to create a confluent monolayer. The cell monolayer was scraped in a straight line to create a “scratch” with a p200 pipet tip. After cells were washed once with the growth medium, the RPMI1640 medium containing 2% FBS was added. The cells were treated with N-Shh conditional medium plus GANT61 or DMSO for 36 hr. And the cells were photographed through a phase-contrast microscope (10× objective lens) at 0, 12, 24 and 36 hr. The wound area was quantified with NIH Image-Pro Plus software. The data are expressed as the means of three independent experiments ± standard deviation.

Cell invasion assay was performed in Transwell plates (8-µm pore size, 6.5-mm diameter; Corning Life Sciences, Lowell, MA) precoated with Matrigel Basement Membrane Matrix (1 mg/ml; BD Biosciences, Franklin Lakes, NJ) according to the manufacturer's protocol. Briefly, highly invasive ovarian cancer SKOV3 cells (5×10^4^ cells/well) were plated to the upper chamber of the system and cultured with RPMI1640 supplemented with 2% FBS. Bottom wells in the system were filled with 500 µl N-Shh conditional medium supplemented with 2% FBS. Wherever applicable human integrin β4 (ITGB4) antibody or control IgG (25 µg/ml) was added to the top chamber, respectively. After 24 hr of incubation, noninvaded cells in the upper chamber were removed, and the cells that had invaded through Matrigel matrix membrane were stained with crystal violet for 30 min. The number of invading cells were counted under inverted microscope (10× objective lens) and photographed.

### cDNA microarray analysis

SKOV3 cells were treated with either GANT61 (20 µM for 60 hr) or control vehicle DMSO. Total RNA from each sample was isolated by TRIzol (Invitrogen) according to the manufacturer's instructions. Double-stranded cDNA and biotin-labeled cRNA were synthesized from RNA (300 ng) using an Illumina® TotalPrep RNA Amplification Kit (Ambion Inc., Austin, TX) according to the manufacturer's protocol. Each biotinylated cRNA (750 ng) was hybridized to an Illumina Human HT expression BeadChip V4 (Illumina Inc., San Diego, CA). Following standard washing steps and staining with Streotavidin-Cy3, the arrays were scanned using an Illumina® BeadArray Reader. Genes with a *DiffScore* less than −20 or more than 20 (i.e. *P*-value<0.01) were considered the differentially expressed genes (DEGs). All raw data are available at GEO accession no. GSE53464. DEGs enrichment analyses were performed using the databases GO (Biological Process) and KEGG pathways [74].

### Real-time PCR

Total RNA (1 µg) was employed to prepare cDNA *via* reverse transcription using cDNA synthesis kit (Invitrogen) according to manufacturer's instructions and analyzed using an Applied Biosystems 7500 PCR Detection System (Applied Biosystems Inc.). Real-time PCR reaction conditions were as follows: activation at 95°C for 10 min with 40 cycles of denaturation at 95°C for 15 s, primer annealing and extension at 60°C for 1 min and ramping back to 95°C. mRNA expression levels of target genes were normalized to the expression of GAPDH and quantified using the comparative CT method. Real-time PCR for each gene was determined in triplicate, and each experiment was repeated at least twice to ensure quantitative accuracy. The primers used for real-time PCR are listed in **Table 1**.

### Xenografted tumor model and antitumor effect of GANT61 *in vivo*

Effects of Gli inhibition on the growth of human ovarian cancer cells were investigated in an s.c. xenograft tumor model [29], [75]. SKOV3 cells (2×10^7^) were implanted s.c. into the flank of BALB/c nu/nu mice. Tumors were grown until they reached a median size of 100 mm^3^, then the mice were randomly assigned to receive either GANT61 (25 mg/kg s.c.) in solvent (corn oil: ethanol, 4∶1) or an equal volume of solvent only for 15 days (treatment scheme according to Figure 5) [29], [75]. The s.c injections were initiated four days after tumor inoculation, and repeated every other day. Tumor size was measured with calipers, and tumor volume was calculated by the formula of length×width×0.5×(length+width) [29]. When the experiment was terminated, s.c. tumors were excised, weighed, and prepared for immunohistochemical analyses. Western blot analyses on tumor tissues were done by using lysis buffer for protein extraction and subsequent SDS-PAGE as described above. This study was carried out in strict accordance with the recommendations in the Guide for the Care and Use of Laboratory Animals of the National Institutes of Health. The protocol was approved by the Committee on the Ethics of Animal Experiments of the First Affiliated Hospital of Nanchang University (Permit Number: 2011-021). All surgery was performed under sodium pentobarbital anesthesia, and all efforts were made to minimize suffering.

### Accession numbers

The GEO accession number for the microarray gene expression data reported in this paper is GSE53464.

### Statistical analysis

Unless otherwise indicated, data were expressed as mean ± SD for experiments performed at least three times. The difference between groups was assessed by Student's *t*-test or one-way ANOVA. Differences were considered significant if *P*<0.05. All analyses were carried out by the use of SPSS13.0 software (SPSS Inc., Chicago, IL).
